# The Presence of Ultra-Traces of Persistent Organic Pollutants (POPs) and Heavy Metals in Some Areas of Molise: The Importance of a “Blank” in Public Health Studies

**DOI:** 10.3390/toxics11030250

**Published:** 2023-03-07

**Authors:** Ivan Notardonato, Francesca Fantasma, Pamela Monaco, Cristina Di Fiore, Gabriella Saviano, Carmen Giancola, Pasquale Avino, Vincenzo De Felice

**Affiliations:** 1 Department of Agriculture, Environmental and Food Sciences, University of Molise, Via De Sanctis, I-86100 Campobasso, Italy; 2 Department of Biosciences and Territory, University of Molise, C. da Fonte Lappone, I-86090 Pesche, Italy; 3 Institute of Atmospheric Pollution Research (IIA), National Research Council (CNR), Rome Research Area-Montelibretti, I-00015 Monterotondo Scalo, Italy

**Keywords:** pollution, air, atmosphere, PAHs, heavy metals, vulnerable populations, exposure, public health

## Abstract

The emission of chemicals into the environment has increased in a not negligible way as a result of the phenomenon of globalization and industrialization, potentially also affecting areas always considered as “uncontaminated”. In this paper, five “uncontaminated” areas were analyzed in terms of the presence of polycyclic aromatic hydrocarbons (PAHs) and heavy metals (HMs), comparing them with an “environmental blank”. Chemical analyses were carried out using standardized protocols. The ‘environmental blank’ results revealed the presence of Cu (<64.9 μg g^−1^), Ni (<37.2 μg g^−1^), and Zn (<52.6 μg g^−1^) as HMs and fluorene (<17.0 ng g^−1^) and phenanthrene (<11.5 ng g^−1^) as PAHs. However, regarding the results of the pollution status of the areas under study, fluorene (#S1, 0.34 ng g^−1^; #S2, 4.3 ng g^−1^; #S3, 5.1 ng g^−1^; #S4, 3.4 ng g^−1^; #S5, 0.7 ng g^−1^) and phenanthrene (#S1, 0. 24 ng g^−1^; #S2, 3.1 ng g^−1^; #S3, 3.2 ng g^−1^; #S4, 3.3 ng g^−1^; #S5, 0.5 ng g^−1^) were found in all areas, while the other PAHs investigated were detected at a concentration averaging less than 3.3 ng g^−1^. HMs were found in all of the investigated areas. In particular, Cd was detected in all areas with an average concentration of less than 0.036 μg g^−1^, while Pb was absent in area #S5, but present in the other areas with an average concentration of less than 0.018 μg g^−1^.

## 1. Introduction

The environment has been significantly affected by human activities, which can be considered as the main source of pollutant emissions. Particularly, among the different human activities, the construction of the urban environment and industrial elements has contributed to the release of high concentrations of heavy metals (HMs) and polycyclic aromatic hydrocarbons (PAHs) into the environment [1,2]. The reduction of so-called “green areas” for “grey areas” has also occurred in Italy, particularly in the last few decades. According to the ISTAT data from 2016, in 111 provincial capitals, urban greenery accounts for only 2.7% of the entire territory [3]. A multitude of chemicals are released into the environment, seriously affecting ecological equilibrium and human health [4]. The scientific literature has reported that, on a daily basis, significant release of persistent organic pollutants (POPs) and heavy metals occurs in several areas of Italy. For example, concentrations exceeding the legal limit values of mercury as well as the high concentration of PAHs have been detected in Augusta-Melilli-Priolo (Sicily, South of Italy) [5].

Significant concentrations of PAHs were also detected in Monopoli and Torre a Mare (Puglia, Southern Italy) [6]. In addition, an article by Imperato and collaborators revealed a pollution phenomenon in Naples (Campania, Southern Italy), where a high accumulation of MM in soils was found [7]. Recent scientific evidence has, in fact, shown a significant accumulation of HMs in both urban and rural areas in Naples. In rural areas, the main contribution is agricultural activities. The experimental data, in fact, underline the not negligible impact of the activities of fertilization with phosphates that have released not negligible concentrations of Cd [8]. On the other hand, urban and industrial areas have been affected by increased levels of vehicle traffic and industrial activities. Experimental data suggest that levels of POPs are higher near heavy traffic roads, next to the railway station, bus stations, and commercial port, underlining the strong impact of human activities on pollution phenomena [9].

Scientific based-research evidence proved that in the last 100 years, heavy metals and PAHs concentrations have, in fact, increased in all biosphere environmental media as a direct response of human activities [10]. Furthermore, the co-contamination of HMs and PAHs has been widely demonstrated and their co-existence is a matter of a great concern due to their synergistic cytotoxic effects [11,12]. Effects on humans due to an acute and/or chronic exposure to these environmental chemicals have been investigated. Heavy metals and PAHs have been, in fact, associated with a wide range of adverse effects such as illness (i.e., cancer, cardiovascular, nervous, kidney, and bone disease) and death [13]. Reactive oxygen species (ROS) levels can significantly increase as a response to heavy metals and PAH exposure. ROS can thus lead to oxidative stress and oxidative DNA damage, which basically consist of the formation of lesions to DNA filaments [14]. However, the effects of exposure to heavy metals and PAH may be different, potentially depending on the health status of the individual [15]. Therefore, it is possible to assume that an exposure may induce different effects, more or less severe, depending on the health status of the individual. A class of population that should be particularly monitored is that defined as “vulnerable”, as it is at risk of poor physical, physiological, and/or social health. There is a lack of research on the effects of heavy metals and PAHs on vulnerable populations and data about their exposure are very limited [16]. Vulnerable people, in fact, can also be seriously affected by low levels of pollutants [17]. The so-called “poorly contaminated” areas are generally characterized by low-levels of contamination; as it is not possible to exclude a priori the impact of low concentrations of contaminants in the environment on specific classes of population (i.e., vulnerable population such as elderly, children, low-income families, newcomers, and ethnic minorities) [17,18], these areas should be carefully monitored.

The purpose of this work is to examine the levels of contamination by POPs such as polycyclic aromatic hydrocarbons (benzo[a]pyrene, bap; benzo[a]anthracene, Baa; benzo[k]fluoranthene, BkFA; chrysene, CHR; Indeno[1,2,3-cd]pyrene, IP; pyrene, PY; benzo[b]fluoranthanthene, BbFA; benzo[g,h,i]perylene, BghiP; dibenzo[a,h]anthracene, DhA) and inorganic pollutants such as heavy metals (i.e., Cd, Pb, Cu, Zn, and Ni) in different areas of the Molise region characterized by pollution sources of different degrees. In addition, a large area of the region considered as uncontaminated has been taken as a reference to assess the background levels of contamination. The study was conducted through a basic regional approach. Indeed, several studies have been carried out on the contamination phenomena in Italy, but most of them have been carried out on a local or small scale, while a basic regional approach is still rare [19].

The Molise region is characterized by low levels of contamination, but the monitoring of contamination levels should be a necessity to protect vulnerable people. As the levels of contamination in the Molise area is low, to provide precise data about the pollutant levels and to distinguish between natural and anthropogenic sources, an “environmental blank” was determined. In this work, the trace levels of contaminants in five areas of Molise were determined. To achieve this purpose, a “non-contaminated” area of Molise was chosen as an “environmental blank”. Analysis for the determination of contamination levels of the blank was performed on a soil sample as it is a major reservoir of heavy metals [11,12]. Instead, the determination of the contamination levels of areas under investigation was performed using honeybees, which have been used as bioindicators of the levels of pollution of the investigated areas. The reason why bees have been used as indicators of pollution status is based on the appropriateness of these living organisms to capture and trap pollutants in their bodies, as demonstrated in the scientific literature [20,21,22].

Finally, based on the scientific information, an assessment about the potential effects on vulnerable populations was carried out.

## 2. Materials and Methods

### 2.1. Study Sites and Sampling for Environmental Blank Analysis

**Figure 1:**
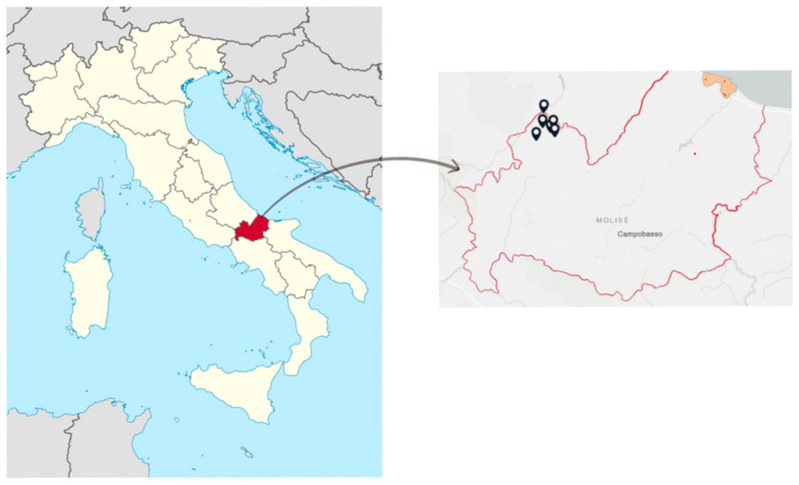
Geographical location of the sites used as an environmental blank in the Molise region (black) and areas under investigation (orange), Italy. The first photo was taken from Google Images, while the indication of the sampling sites was made through ArcGIS Online. Geographical coordinates of sites: Monteforte (41.797489, 14.291578, Molise region), Verrino (41.816970, 14293321, Molise region), Guardata (41.847971, 14.277226, Molise region), Guado Cannavina (41.849737, 14.337791, Molise region), Macchia Bassa (41.840580, 14.333817), Monte San Nicola (41.845736, 14.327491), urban center of Termoli (#S1) (41.993099, 14.988218), industrial area of Termoli (#S2–#S3) (41.949167, 14.989022), rural area of Termoli (#S4) (41.9955667, 14.956600), and natural reserve of Termoli (#S5) (41.722638, 14.871389).

Soil samples for the environmental blank analysis were collected from six different sites of a rural area of the Molise region (Central of Italy), which are indicated in black on the map (Figure 1). Figure 1 also shows the five areas investigated to study the levels of contamination in the Molise region (in orange).

The six locations surveyed had a total extension of about 0.960 km^2^. Two representative sites were identified for each of the six locations. Systematic sampling was carried out at each site, drawing two squares (5 × 5 m^2^). The herbaceous cover of each square was carefully removed. For each square, five soil aliquots were taken, then sieved on the spot with a sieve with large meshes (1 cm) and mixed. In this way, for each locality, 20 soil samples were taken, reduced to two representative samples for each locality. The total number of samples in the whole sample area was 12. Therefore, the soil samples were transported to the chemistry laboratory of the University of Molise for the chemical analysis.

### 2.2. Study Sites and Sampling for HMs and PAHs Analysis

For the determination of the pollution levels in the Molise region, five areas were taken into account with different levels and sources of contamination. Area 1 is an urban center of Termoli, where one monitoring station (#S1) was positioned. This area is characterized by a significant intensity of vehicular traffic. Area 2 is an industrial area, where two monitoring stations (#S2–#S3) were positioned, as the number of stations was established according to the extension of the territory to be monitored. The industrial area has food, chemical, and metal industries, which are a significant source of pollutants. Station #S4 was positioned in a rural area. The rural area of Termoli and its surroundings is characterized by intense agricultural activity using agricultural machinery. The area was selected on the basis of the impact that rural activities have on the release of pollutants. Station #S5 was placed at the Lipu Oasis, an area known to be sparingly polluted. However, this area is used for recreational activities (i.e., barbeques) that may have an impact, albeit minimal, on the release of pollutants.

Sampling of HMs and PAHs was conducted collecting honeybees. Sampling lasted for about 7 months (March-October), and during it, no chemicals were used to treat the honeybees to not invalidate the results. After collection, the bee samples were stored in the freezer at −20 °C.

### 2.3. Extraction and Determination of HMs from Soil Samples

For the extraction of heavy metals (i.e., Cd, Pb, Zn, Ni and Cu), 0.5 ± 0.05 g of soil was processed by means of a digester tube using 3 mL of hydrogen peroxide to remove the organic matter. Soil samples were then mineralized with 9 mL of hydrochloric acid (Baker Instra-Analyzed, Fisher Scientific, Waltham, MA, USA) and 3 mL of nitric acid (68%) for 40 min at 180 °C and then filtered by means of Whatman no. 42 filters (pore size: 2.5 µm). An atomic absorption spectrophotometer (SpectrAA 220 FS, Varian, Santa Clara, CA, USA) was used for the determination of heavy metals. The concentrations of heavy metals in the soil samples were determined by means of calibration curves, and procedural blanks were used to ensure the absence of contamination in the laboratory. Calibration curves were prepared by diluting a multi-Element standard mix of heavy metals in 10% HNO_3_ (O2Si Smart Solutions, North Charleston, SC, USA).

The chemical analyses were carried out in triplicate.

### 2.4. Extraction and Determination of PAHs from Soil Samples

To determine the PAHs, the soil samples were extracted by means of a Soxhlet extractor. In brief, 10 ± 0.05 g of soil was mixed with 10 g of sodium sulfate anhydrous and then placed into a cellulose thimble. Thus, the soil samples were dived in an acetone–hexane mixture (1 + 1, *v*/*v*) at 140 °C for 60 min. After, the soil samples were washed with the solvent mixture vapors for 60 min. The extraction solvent was then recovered and dried under a gentle flow of nitrogen in a fume hood. Analytes were then recovered with 1.0 mL of *n*-hexane and an appropriate volume of the experimentally determined internal standard (octacosane, C_28_H_58_) was added. Then, 1 μL was injected into the gas chromatograph-mass spectrometer (gas chromatograph, Trace 1310, equipped with a triple quadrupole mass spectrometer, QqQ, 7000D, Agilent Technologies, Santa Clara, CA, USA) for the analysis. Quantification was carried out by means of calibration curves. Calibration curves were prepared by diluting a standard solution of PAHs in methanol/dichloromethane (1 + 1 *v/v*) (Certified Reference Material, CAPChem Ltd., Belgium). The chemical analyses were carried out in triplicate.

### 2.5. Extraction and Determination of HMs from Honeybee Samples

For the analysis of HMs from the honeybee samples, 4 g of each bee sample was placed into a porcelain capsule and then in a muffle at 130 °C. After each hour, the temperature of the muffles was increased up to 380 °C (50 °C every hour). Afterward, the samples were left to cool and placed into a flacon (50 mL), and 5 mL of nitric acid (5%) was used to recover the samples. Finally, the samples were transferred into the ED36S Digiblock digester (LabTech, Boston, MA, USA) for 30 minutes at 120 °C. After cooling, Milli-Q ultrapure water was added to each sample up to a volume of 25 mL and analyzed by means of an ICP-OES (5110 Agilent Technologies, Santa Clara, CA, USA). Wavelengths used for each analyte for ICP-OES analysis were as follows: Zn 213.857 nm, Cd 228.802 nm, Pb 405.781 nm, Ni 361.939, and Cu 324.754 nm.

Calibration curves were prepared by diluting a multi-element standard mix of heavy metals in 10% HNO_3_ (O2Si Smart Solutions, USA). The chemical analyses were carried out in triplicate.

### 2.6. Extraction and Determination of PAHs from Honeybee Samples

Briefly, 5 g of shredded honeybee samples were mixed with anhydrous sodium sulfate and diatomaceous earth and transferred into the cells for the accelerated solvent extraction technique (ASE). Cellulose filters were used to pack the cells and then they were placed into the ASE system. A mixture of dichloromethane/acetone (1 + 1, *v/v*) was used as the extraction solvent with the following extraction parameters: temperature was set to 150 °C, pressure to 2000 psi kept constant through a nitrogen flow for each extraction cycle. At the end of the extraction cycle, each organic extract was collected in a vial and then transferred into a glass flask for evaporation, using filter paper with hygroscopic sodium sulfate and Florisil in order to remove any possible traces of water in the extract. The solvent was then dried using a rotavapor R-200 BÜCHI at a temperature up to 40 °C and the extract was dissolved in cyclohexane (500 μL). Afterward, solid phase extraction (SPE) was used to clean up the samples. Prior to use, SPE cartridges were rinsed with ethyl acetate (6 mL) and cyclohexane (6 mL). The sample was placed into the SPE system for cleaning up and the elution was obtained by using cyclohexane (6 mL). The eluate was thus collected in a vial and concentrated up to 500 μL under a gentle nitrogen flow. A total of 1 μL of the solution was used for the analysis, which was conducted using a GC (Trace 1310) equipped with a triple quadrupole mass spectrometer detector (GC/QqQ 7000D, Agilent Technologies).

## 3. Results

### 3.1. Soil Samples Results: Environmental Blank

The results on the HMs and PAHs obtained from the analysis of soil samples collected from the six localities of the Molise region are reported in Table 1 and Table 2.

As shown in Table 1, only three of the five heavy metals investigated were found in the study areas. Particularly, Cu, Ni, and Zn were detected. Cu was present with an average concentration of 43.6 μg g^−1^, Ni in an average concentration of 20.1 μg g^−1^, and Zn in an average concentration of 40.6 μg g^−1^. Cd and Pb were below the LOD values. Concerning the PAHs, of the 18 PAHs investigated, only fluorene and phenanthrene were revealed. Particularly, phenanthrene was greater than the LOD value only in Verrino and Guado Cannavina, whereas fluorene was detected in all areas under study, except for Monteforte. Particularly, the highest value was detected in Guardata (17.0 ng g^−1^), whereas the lowest one was in Macchia Bassa and Verrino (2.5 ng g^−1^). The analysis of the “environmental blank” showed that both PAHs and HMs detected were under the legal limits established by Italian environmental law (Legislative Decree No. 152/2006), which regulates the threshold values of some contaminants in the soil [23]. The LOD values for each parameter studied are reported in Table 3.

### 3.2. Honeybee Sample Results

**Figure 2:**
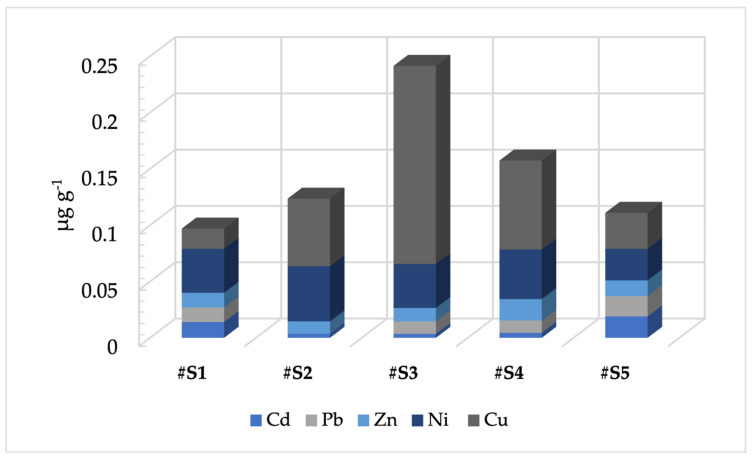
Levels of HMs in the five areas of the Molise region under study during the sampling period from March to October. For sampling areas: see the text.

Honeybee samples were used to analyze the levels of HMs in the five areas of the Molise region under study. The results obtained are shown in Figure 2.

The HM levels detected by means of honeybees were lower than those found in the areas considered as the “environmental blank” for Zn (#S1 0.013; #S2 0.011; #S3 0.012; #S4 0.019; #S5 0.014 μg g^−1^), Cu (#S1 0.018; #S2 0.060; #S3 0.176; #S4 0.079; #S5 0.032 μg g^−1^), and Ni (#S1 0.039; #S2 0.049; #S3 0.039; #S4 0.044; #S5 0.028 μg g^−1^). Pb and Cd were not detected in the areas of the “environmental blank”, but they were in the areas under study. Particularly, Cd was revealed in the following concentrations: 0.014 μg g^−1^ for #S1, 0.0036 μg g^−1^ for #S2, 0.0035 μg g^−1^ for #S3, 0.0044 μg g^−1^ for #S4, and 0.019 μg g^−1^ for #S5. Likewise, Pb was not detected in the environmental blank area, but it was present in the following concentrations in the areas under study: 0.013 μg g^−1^ for #S1, 0.011μg g^−1^ for #S3, 0.01 μg g^−1^ for #S4 and 0.018 μg g^−1^ for #S5, and below the LOD value in #S2.

**Figure 3:**
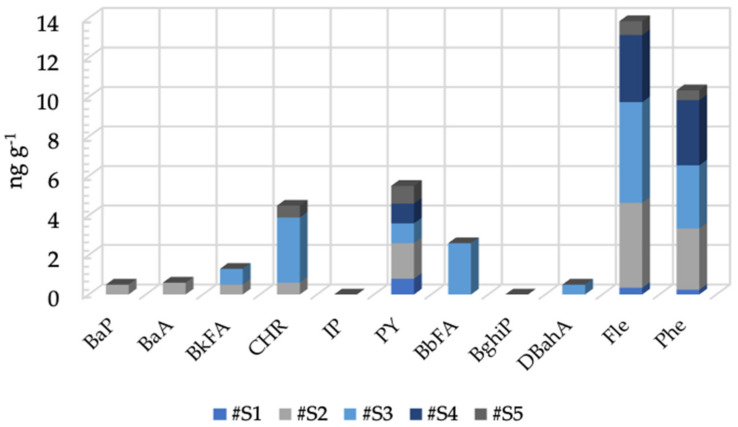
Levels of PAHs in the five areas of the Molise region under study during the sampling period from March to October. For sampling areas: see the text.

The PAH levels detected by means of honeybee were lower than those found in the areas considered as the “environmental blank” for Fle (#S1 0.34; #S2 4.3; #S3 5.1; #S4 3.4; #S5 0.7 ng g^−1^) and Phe (#S1 0.24; #S2 3.1; #S3 3.3; #S4 3.2; #S5 0.5 ng g^−1^) (Figure 3). All of the other PAHs investigated were not detected in the areas considered as the environmental blank, but they were present in some areas under investigation. Particularly, BaP, BaA, BkFA, CHR, PY, Fle, and Phe were detected in #S2 in concentrations of 0.5, 0.6, 0.5, 0.6, 1.8, 4.3, and 3.1 ng g^−1^, respectively. PY, Fle, and Phe were revealed in area #S1, in concentrations of 0.8, 0.34, and 0.24 ng g^−1^, respectively. Area #S3 was characterized by the presence of BkFA, CHR, PY, BbFA, DBahA, Fle, and Phe in the following concentrations: 0.8, 3.3, 1.0, 2.6, 0.5, 5.1, and 3.2 ng g^−1^, respectively. Area #S4 was contaminated by PY, Fle, and Phe in the following concentrations: 1.0, 3.4, and 3.3 ng g^−1^, respectively. In area #S5, CHR, PY, Fle, and Phe were detected in the following concentrations: 0.6, 0.9, 0.7, and 0.5 ng g^−1^.

## 4. Discussion

### 4.1. Environmental Contamination Levels

In this paper, an analysis of the levels of contamination in some areas of Molise was carried out. Basically, the main purpose of the work was to assess whether, even in areas known to be “poorly polluted”, they are affected by the presence in traces or ultra-traces of PAHs and MHs that can have adverse effects on human health. According to the scientific literature, the assessment and determination of low levels of contamination is necessary to understand, and understand in depth, the metabolic effect that may occur in humans after exposure to low concentrations, but persistent over time, to pollutants [11].

As a matter of fact, the evaluation of the effects on humans after exposure to pollutants can be divided in two steps: the first step consists of a screening process, which aims to collect and analyze data on site-specific contamination levels. The second step focuses on characterizing the effects of contaminants, usually based on the literature, trying to employ conservative assumptions [24].

First, an environmental blank was taken into account to clearly understand the background contamination value of the areas under investigation. Hence, the first step of the screening process was carried out by determining the environmental blank and the contamination levels of the five areas under study.

The analysis of the environmental blank showed that only fluorene and phenanthrene were detected in low concentrations. Based on the scientific knowledge, fluorene and phenanthrene are considered as the two prevalent PAHs as they have been detected in various areas [25]. However, the contamination by fluorene and phenanthrene can be due to road surface renovation works carried out a few days before the start of the sampling. Furthermore, the environmental blank area was located next to rural areas. Hence, the agricultural vehicles released PAHs into the environment as the combustion of fossil fuels is one of the main sources [25]. In addition, their hydrophobic nature and their preference to be adsorbed by solid matrices makes them capable of accumulating in the soil [26]. Therefore, it is plausible to hypothesize that their presence in the area is not due to the presence of a fixed source, but to spot events.

Areas under study presented low levels of contamination. PAHs were detected in all five areas investigated. The lowest levels were found in areas #S1 and #S5, whereas an increase in the PAH concentration values were observed in areas #S2, #S3, and #S4. However, considering the contamination levels of other rural areas in Italy, values found in the Molise region were much lower. For example, rural areas of the Campania region showed a PAH concentration between 276 and 11,353 ng g^−1^; the Sicily countryside also showed PAH concentrations within 52.45–74.25 ng g^−1^. Another study conducted in soils from Naples identified as the most present PAHs were fluorene, pyrene, and chrysene [27]. This is consistent with the results obtained in the present study. Italian urban areas are characterized by high levels of PAHs. More specifically, Rome showed a PAH concentration of 755 ng g^−1^, which was close to the values detected in Naples of 715 ng g^−1^ [19].

The levels of HMs detected in the environmental blank area were all below the legal limits established by the Italian Environmental law for a public or private “green” area (e.g., Ni, 120 μg g^−1^, Cu, 120 μg g^−1^ and Zn 150 μg g^−1^, Cd, 2 μg g^−1^, Pb, 100 μg g^−1^). HMs were detected in the five areas of Molise. More specifically, Cd and Pb were detected in all areas under study, except for area #S2, where Pb was not revealed. Among the HMs, Cu was present in the highest concentrations. Fertilizers used in the sampled areas can determine an increase in the Cu concentration [20]. The levels of pollutants tend to vary according to the anthropic characteristics of the area considered. A work by Perna and co-authors, in fact, showed a statistically significant difference of some heavy metals between a rural and an urban area. Differences in the toxic element content between areas are due to environmental factors with an important contribution of the anthropic impact typology [28]. In our study, the anthropic impact was mainly found in the presence of Cu. Basically, all areas analyzed in this study are surrounded by farmland and the use of Cu as a fertilizer is quite widespread. As for the Pb, Zn, and Cd found in the #S1 area, an important source of contamination is the train station. Scientific literature reveals that friction processes between wheelsets and rails during rail transport cause railways to release heavy metals including Zn, Cd, and Pb into the environment [29,30]. The industrial area (#S2–#S3) is characterized by industries of different types. However, of greater importance, by size, is the metallurgical industry. The emissions of Pb, Cd, and Ni are due to the metallurgical industry, active for years in the area examined [31,32]. Unexpectedly, area #S5, considered as a little contaminated area, showed the presence of Pb, Cd, Ni, Zn, and Cu, with a higher average concentration than area #S1. Despite the low industrialization of the #S5 area, human activities (i.e., railway passage, agricultural activities) [33] are sufficient to release heavy metals.

However, it is worth noting that it is currently not possible to define whether the PAH and HM values found in the five areas under study (i.e., #S1, #S2, #S3, #S4, and #S5) were below the values considered as tolerable by law. Both the Italian and European legislation, in fact, do not provide limits of pollutants for sampling carried out through the use of living organisms (e.g., bee) [34]. For this reason, if it is possible to define that the values of the “environmental white” are below the legal limit established by the Environmental Italian Law (Legislative Decree No. 152/2006), given that the determination was carried out on soil samples, and no formally recognized legal limits are available for bees.

### 4.2. Exposure to Environmental Chemical of the Vulnerable Populations

Exposure to environmental chemicals is a topic of concern, as different toxic effects can be associated with them. Endocrine related cancers, obesity, type 2 diabetes, immune related disorders, decrease in fertility, and adverse pregnancy outcomes are some examples of endocrine related disorders resulting from exposure to chemicals [35]. Environmental chemicals affect vulnerable populations, particularly with regard to pregnant women, children, toddlers, and individuals with low socioeconomic status [36]. Among them, fetus health status is often considered as an important segment of the vulnerable population [35]. Research by Karttunen et al. (2010) showed that maternal exposure to BaP led to exposure of the fetus to it and/or its metabolites. Indeed, the use of the human placenta perfusion allowed us to understand that a concentration from 0.1 to 1 μM of BaP is able to reach the fetal compartment. Furthermore, the concentrations of BaP used simulated the exposure of the pregnant woman and fetus to low levels of contaminants, underlying the impact on a vulnerable segment of populations to low levels of pollutants [37]. PAHs and HMs also showed a negative effect on vulnerable populations; furthermore, it has been reported that vulnerable populations tend to be more exposed to environmental chemicals. For example, another important segment of vulnerable populations are low-income families as they are more exposed to contaminants that come from industrial areas, but environmental policies do not seem to protect these populations [18]. For example, due to different behavioral habits (i.e., hand-finger sucking), children are more exposed to heavy metals [38].

## 5. Conclusions

The present work aimed to identify the trace and ultra-trace levels of PAH and HM in five areas of the Molise region, comparing them with the concentrations of the same present in a notoriously “non-polluted” area and considered as an “environmental blank”. In the first place, the identification of an “environmental blank” is essential to correctly define the pollution levels of the area. The sampling of the area identified as “blank” was carried out on soil samples, both to be able to define the pollution status in relation to the reference legislation, and because the soil is the main sink for pollutants. The five areas studied instead showed the presence of PAHs and HMs in traces, due to anthropic agricultural and industrial activities. Although the level of pollution is low, the continuous presence of pollutants over time could, in the long run, be harmful to vulnerable individuals. In conclusion, this work highlights the need to carefully and continuously monitor areas that can be considered as little exposed to contamination phenomena, and therefore as “green areas”. In fact, the experimental data collected can provide useful information for the implementation of measures aimed at protecting the state of health of more vulnerable individuals.

## Figures and Tables

**Table 1:** Results of the HMs (μg g^−1^) detected in the six localities under investigation for the analysis of the environmental blank. *: for each HM, the legal limits (expressed as μg g^−1^) from the Italian Environmental Law (Legislative Decree No. 152/2006) are reported.

Locality	Cu	Cu *	Ni	Ni *	Zn	Zn *
Monteforte	61.0 ± 2.1	120	37.2 ± 5.6	120	52.6 ± 3.4	150
Verrino	27.2 ± 0.8		14.1 ± 0.5		39.7 ± 3.2	
Guardata	21.6 ± 0.3		12.9 ± 1.6		46.5 ± 7.4	
Guado Cannavina	22.7 ± 1.7		24.3 ± 2.8		38.8 ± 2.2	
Monte S. Nicola	64.2 ± 1.8		16.3 ± 1.4		34.2 ± 5.1	
Macchia Bassa	64.9 ± 2.4		16.5 ± 0.9		31.9 ± 2.8	

**Table 2:** Results of the PAHs (ng g^−1^) detected in the six localities under investigation for the analysis of the environmental blank. *: for each PAH, the legal limits (expressed as μg g^−1^) from the Italian Environmental Law (Legislative Decree No. 152/2006) are reported.

Locality	Fluorene	Fluorene *	Phenanthrene	Phenanthrene *
Monteforte	<LOD	50	<LOD	50
Verrino	2.5 ± 0.7		9.5 ± 0.4	
Guardata	17.0 ± 0.4		<LOD	
Guado Cannavina	15.0 ± 0.9		11.5 ± 0.8	
Monte S. Nicola	4.0 ± 0.5		<LOD	
Macchia Bassa	2.5 ± 0.05		<LOD	

**Table 3:** Instrumental values of LODs for all PAHs (ng g^−1^) analyzed by a GC-MS (QqQ) and HMs (μg g^−1^), analyzed by means of an atomic absorption spectrophotometer and an ICP-OES.

HMs	ICP-OES	AA
Cu	0.05	4.0
Zn	0.02	3.0
Pb	0.01	1.0
Cd	0.02	2.0
Ni	0.02	3.0
**PAHs**	**GC-MS (QqQ)**	
BaP	0.02	
BaA	0.02	
BkFA	0.03	
CHR	0.03	
IP	0.05	
PY	0.04	
BbFA	0.05	
BghiP	0.06	
DBahA	0.06	
Fle	0.07	
Phe	0.07	

## Data Availability

The data presented in this study are available on request from the corresponding author.

## References

[1] Armiento G., Caprioli R., Cerbone A., Chiavarini S., Crovato C., De Cassan M., De Rosa L., Montereali M.R., Nardi E., Nardi L. (2020). Current status of coastal sediments contamination in the former industrial area of Bagnoli-Coroglio (Naples, Italy). Chem. Ecol..

[2] Cilluffo G., Ferrante G., Fasola S., Montalbano L., Malizia V., Piscini A., Romaniello V., Silvestri M., Stramondo S., Stafoggia M. (2018). Associations of greenness, greyness and air pollution exposure with children’s health: A cross-sectional study in Southern Italy. Environ. Health.

[3] WWF Alarm: City Less and Less Green. https://www.corriere.it/ambiente/18_ottobre_06/wwf-verde-urbano-citta-italiane-8dca0dfa-c9a0-11e8-9bde-b14535fa581c.shtml.

[4] Wahlang B. (2018). Exposure to persistent organic pollutants: Impact on women’s health. Rev. Environ. Health.

[5] Di Bella C., Traina A., Giosuè C., Carpintieri D., Lo Dico G.M., Bellante A., Del Core M., Falco F., Gherardi S., Uccello M.M. (2020). Heavy metals and PAHs in meat, milk and seafood from Augusta Area (Southern Italy): Contamination levels, dietary intake and human exposure assessment. Front. Public Health.

[6] Mali M., Dell’Anna M.M., Mastrorilli P., Damiani L., Piccinni A.F. (2017). Assessment and source identification of pollution risk for touristic ports: Heavy metals and polycyclic aromatic hydrocarbons in sediments of 4 marinas of the Apulia region (Italy). Mar. Pollut. Bull..

[7] Imperato M., Adamo P., Naimo D., Arienzo M., Stanzione D., Violante P. (2003). Spatial distribution of heavy metals in urban soils of Naples city (Italy). Environ. Pollut..

[8] Vingiani S., De Nicola F., Purvis W.O., Concha-Grana E., Muniategui-Lorenzo S., Lopez-Mahia P., Giordano S., Adamo P. (2015). Active Biomonitoring of Heavy Metals and PAHs with Mosses and Lichens: A Case Study in the Cities of Naples and London. Water Air Soil Pollut..

[9] Golia E.E., Papadimou S.G., Cavalaris C., Tsiropoulos N.G. (2021). Level of Contamination Assessment of Potentially Toxic Elements in the Urban Soils of Volos City (Central Greece). Sustainability.

[10] Cristaldi A., Olivieri Conti G., Jho E.H., Zuccarello P., Grasso A., Copat C., Ferrante M. (2017). Phytoremediation of contaminated soils by heavy metals and PAHs. A brief review. Environ. Technol. Innov..

[11] Ali M., Song X., Ding D., Wang Q., Zhang Z., Tang Z. (2022). Bioremediation of PAHs and heavy metals co-contaminated soils: Challenges and enhancement strategies. Environ. Pollut..

[12] Wu S., Zhou B.H., Chen D., Wang C., Li B., Tong G., Yuan Y., Xu B. (2019). Improving risk management by using the spatial interaction relationship of heavy metals and PAHs in urban soil. J. Hazard. Mater..

[13] Ritter L., Solomon K.R., Forget J. (1999). Inventory of Information Sources on Chemicals—Persistent Organic Pollutants.

[14] Xu J., Liu Y., Zhang Q., Su Z., Yan T., Zhou S., Wang T., Wei X., Chen Z., Hu G. (2022). DNA damage, serum metabolomic alteration and carcinogenic risk associated with low-level air pollution. Environ. Pollut..

[15] Aday L.A. (1994). Health status of vulnerable populations. Annu. Rev. Public Health.

[16] Trejo-Acevedo A., Diaz-Barriga F., Carrizales L., Dominguez G., Costilla R., Ize-Lema I., Yarto-Ramirez M., Gavilan-Garcia A., Mejia-Saavedra J.J., Perez-Maldonado I.N. (2009). Exposure assessment of persistent organic pollutants and metals in Mexican children. Chemosphere.

[17] Lee D.H., Steffes M.W., Sjödin A., Jones R.S., Needham L.L., Jacobs D.R. (2010). Low dose of some persistent organic pollutants predicts Type 2 diabetes: A nested case-control study. Environ. Health Perspect..

[18] Montano-Lopez F., Biswas A. (2021). Are heavy metals in urban garden soils linked to vulnerable populations? A case study from Guelph, Canada. Sci. Rep..

[19] Thiombane M., Albanese S., Di Bonito M., Lima A., Zuzolo D., Rolandi R., Qi S., De Vivo B. (2019). Source patterns and contamination level of polycyclic aromatic hydrocarbons (PAHs) in urban and rural areas of Southern Italian soils. Environ. Geochem Health.

[20] Di Fiore C., Nuzzo A., Torino V., De Cristofaro A., Notardonato I., Passarella S., Di Giorgi S., Avino P. (2022). Honeybees as bioindicators of heavy metal pollution in urban and rural areas in the South of Italy. Atmosphere.

[21] Di Fiore C., De Cristofaro A., Nuzzo A., Notardonato I., Ganassi S., Iafigliola L., Sardella G., Ciccone M., Nugnes D., Passarella S. (2023). Biomonitoring of polycyclic aromatic hydrocarbons, heavy metals, and plasticizers residues: Role of bees and honey as bioindicators of environmental contamination. Environ. Sci. Pollut. Res..

[22] Laconi A., Tolosi R., Mughini-Gras L., Mazzucato M., Ferrè N., Carraro L., Cardazzo B., Capolongo F., Merlanti R., Piccirillo A. (2022). Beehive products as bioindicators of antimicrobial resistance contamination in the environment. Sci. Total Environ..

[23] Legislative Decree 3 April 2006, n. 152, Environmental Regulations. https://www.bosettiegatti.eu/info/norme/statali/2006_0152.htm.

[24] Goómez-Gutiérrez A., Garnacho E., Bayona J.M., Albaigés J. (2007). Screening ecological risk assessment of persistent organic pollutants in Mediterranean Sea sediments. Environ. Int..

[25] Salehi S.Y., Deljoo S., Harzandi A.M. (2015). Fluorene and phenanthrene uptake and accumulation by wheat, alfalfa and sunflower from the contaminated soil. Int. J. Phytoremediation.

[26] Rabodonirina S., Rasolomampianina R., Krier F., Drider D., Merhaby D., Net S., Ouddane B. (2019). Degradation of fluorene and phenanthrene in PAHs-contaminated soil using Pseudomonas and Bacillus strain isolated from oil spill sites. J. Environ. Manag..

[27] Qu C., Albanese S., Lima A., Hope D., Pond P., Fortelli A., Romano N., Cerino P., Pizzolante A., De Vivo B. (2019). The occurrence of OCPs, PCBs, and PAHs in the soil, air, and bulk deposition of the Naples Metropolitan area, southern Italy: Implication for sources and environmental processes. Environ. Int..

[28] Perna A., Grassi G., Gambacorta E., Simonetti A. (2021). Minerals content in Basilicata region (southern Italy) honeys from areas with different anthropic impact. Int. J. Food Sci..

[29] Vaiškūnaitė R., Jasiūnienė V. (2020). The analysis of heavy metal pollutants emitted by railway transport. Transport.

[30] Briggs D.J. (2007). The use of GIS to evaluate traffic-related pollution. Occup. Environ. Med..

[31] Islam M.M., Akther S.K.M., Hossain M.F., Parveen Z. (2022). Spatial distribution and ecological risk assessment of potentially toxic metals in the Sundarbans mangrove soils of Bangladesh. Sci. Rep..

[32] Rozanski S., Jaworska H., Matuszczak K., Nowak J., Hardy A. (2017). Impact of highway traffic and the acoustic screen on the content and spatial distribution of heavy metals in soils. Environ. Sci. Pollut. Res..

[33] Kasa E., Felix-Henningsen P., Duering R.A., Gjoka F. (2014). The occurrence of heavy metals in irrigated and non-irrigated arable soils, NW Albania. Environ. Monit. Assess..

[34] Ruiz J.A., Gutiérrez M., Porrini C. (2013). Biomonitoring of bees as bioindicators. Bee World.

[35] Mathiesen L., Buerki-Thurnherr T., Pastuschek J., Aengenheister L., Knudsen L.E. (2021). Fetal exposure to environmental chemicals; insights from placental perfusion studies. Placenta.

[36] Gaston S.A., Birnbaum L.S., Jackson C.L. (2020). Synthetic chemicals and cardio metabolic health across the life course among vulnerable populations: A review of the literature from 2018 to 2019. Curr. Environ. Health Rep..

[37] Karttunen V., Myllynen P., Prochazka G., Pelkonen O., Segerback D., Vahakangas K. (2010). Placental transfer and DNA binding of benzo(a)pyrene in human placental perfusion. Toxicol. Lett..

[38] Chen H., Teng Y., Lu S., Wang Y., Wang J. (2015). Contamination features and health risk of soil heavy metals in China. Sci. Total Environ..

